# Antiarrhythmic Medication in Neonates and Infants with Supraventricular Tachycardia

**DOI:** 10.1007/s00246-022-02853-9

**Published:** 2022-03-08

**Authors:** Diana Bruder, Roland Weber, Matthias Gass, Christian Balmer, Anna Cavigelli-Brunner

**Affiliations:** 1 Pediatric Cardiology, Pediatric Heart Center, Department of Surgery, University Children’s Hospital Zurich, Steinwiesstrasse 75, 8032 Zurich, Switzerland; 2 Children’s Research Centre, University Children’s Hospital Zurich, Steinwiesstrasse 75, 8032 Zurich, Switzerland

**Keywords:** Supraventricular tachycardia, Neonates, Infants, Antiarrhythmic medication

## Abstract

Supraventricular tachycardia (SVT) is the most common arrhythmia in neonates and infants, and pharmacological therapy is recommended to prevent recurrent episodes. This retrospective study aims to describe and analyze the practice patterns, effectiveness, and outcome of drug therapy for SVT in patients within the first year of life. Among the 67 patients analyzed, 48 presented with atrioventricular re-entrant tachycardia, 18 with focal atrial, and one with atrioventricular nodal re-entrant. Fetal tachycardia was reported in 27%. Antiarrhythmic treatment consisted of beta-receptor blocking agents in 42 patients, propafenone in 20, amiodarone in 20, and digoxin in 5. Arrhythmia control was achieved with single drug therapy in 70% of the patients, 21% needed dual therapy, and 6% triple. Propafenone was discontinued in 7 infants due to widening of the QRS complex. After 12 months (6–60), 75% of surviving patients were tachycardia-free and discontinued prophylactic treatment. Patients with fetal tachycardia had a significantly higher risk of persistent tachycardia (*p*: 0.007). Prophylactic antiarrhythmic medication for SVT in infancy is safe and well tolerated. Arrhythmia control is often achieved with single medication, and after cessation, most patients are free of arrhythmias. Infants with SVT and a history of fetal tachycardia are more prone to suffer from persistent SVT and relapses after cessation of prophylactic antiarrhythmic medication than infants with the first episode of SVT after birth.

## Introduction

Supraventricular tachycardia (SVT) is the most common arrhythmia in children, occurring with an estimated incidence of 0.1–0.4% in the pediatric population [1–4]. The first episode of an SVT occurs during the first year of life in 50–60%, predominantly in the first 3–4 months [4–7].

The most common paroxysmal SVT in the pediatric age group is atrioventricular re-entrant tachycardia (AVRT), which is mediated by an accessory pathway. This type of tachycardia accounts for 70–80% of tachycardias in infancy. The second most frequent tachycardia, with a much lower incidence in infants (5-17%), is atrioventricular nodal re-entrant tachycardia (AVNRT). Focal atrial tachycardia (FAT) is diagnosed in 5 to 10% of infants with SVT [1–3, 8].

SVT can occur as a unique, sometimes self-limited episode or as incessant and prolonged episodes that can lead to high morbidity without adequate treatment, especially in neonates and infants [2, 5, 9]. After diagnosing a first episode of SVT in an infant, most clinicians have adopted a strategy of giving prophylactic antiarrhythmic drugs for 6 to 12 months [3, 4, 7, 10]. Remarkably, there is neither consensus nor evidence about the optimal approach to the medication or duration of the therapy, and clinical trials about medical treatments are scarce [11, 12]. Recently, it has been suggested that the duration of prophylactic antiarrhythmic medication may be substantially shortened [12]. A large variety of antiarrhythmic drugs is used in neonates and infants [3]: the drugs most commonly used are currently beta-receptor blocking agents, sodium-channel blockers (e.g., flecainide and propafenone), amiodarone, sotalol, and digoxin [3, 4, 6, 11].

In general, the natural history of infant SVT is favorable, with at least 70% of cases resolving spontaneously by 1 year of age [5, 7, 9, 10]. The risk of a recurrence of SVT for infants is about 22–55%, which is lower than in older children [7, 9, 10, 13]. The use of radiofrequency catheter ablation in infants is limited to patients with severe and drug-refractory tachycardias, since the intervention is associated with a lower success rate and more frequent complications in this age group [3, 6, 14]. For children over 15 kg and 4 years of age with persistent symptomatic SVT, elective catheter ablation is a well-established intervention with favorable results [3].

Our experience of patients with incessant tachycardias and/or with frequent relapses has shown that the selection of individual-appropriate antiarrhythmic medication can be challenging and may require dual or triple antiarrhythmic drug therapy. Furthermore, establishing an individual-appropriate medical regimen can take several weeks.

This retrospective study aims to provide critical assessment of drug type and dosage, preferred practice patterns, and patient outcome to define predictors of successful antiarrhythmic medication and risk factors for relapses and/or continuing tachycardias at later follow-up. Our data add evidence about the treatment of SVT in neonates and infants and, thus, contribute to improved overall patient management.

## Methods

This is a retrospective single-center analysis over a ten years period of all consecutive patients who were diagnosed postnatally within the first year of life as having SVT. Patients with a history only of fetal tachycardia without any postpartum SVT were excluded, as were patients with atrial flutter, atrial fibrillation, and relevant structural heart defects. Patients with small, hemodynamic nonsignificant patent arterial duct and atrial or ventricular septal defect remained within the study cohort. The following data were reviewed for each patient: age at first SVT onset, history of fetal tachycardia, initial antiarrhythmic treatment and subsequent antiarrhythmic medications, analysis of 12-lead and 24 h Holter ECGs, echocardiographic parameters of ventricle size and ventricular ejection fraction (EF) with biplane Simpson’s method to document the left ventricular function (defined as EF > 50%: normal, EF 40–50: mildly, EF 30–40 moderately, EF < 30: severely reduced), duration of hospital stay, duration of follow-up, and outcome. The type of SVT was assessed from 12-lead surface ECG; recurrence of tachycardia was defined as an episode of SVT documented on 12-lead ECG, 24 h Holter ECG, or other monitoring device.

Twenty-four-hour Holter ECG monitoring was performed prior to discharge and subsequently at 1 month follow-up and then every 1–3 month according to the clinical state of the patient. With therapy changes it was recorded more often.

At our institution, prophylactic therapy may consist of any of these oral medications: propranolol every 8 h, target doses 3–5 mg/kg/day; propafenone every 8 h, target doses 150–600 mg/m^2^/day; flecainide every 12 h, target doses 3-8 mg/kg/day, amiodarone loading dose of 10–15 mg/kg/day for 10 days in one single dose, then reduction to once a day 5–10 mg/kg/day; and digoxin aiming for a target serum level of 0.6–1.3 nmol/L. Depending on the initial presentation, the medication might be started intravenously and then switched to oral formula.

Beta-receptor blocking agents are the standard first-line treatment, sodium-channel blockers (Class 1C according to the Vaughan-Williams classification) are used particularly for the treatment of FAT or in addition to a beta–receptor blocking agent for patients with ventricular pre-excitation or persistent arrhythmias. Amiodarone is the therapy of choice for patients with impaired ventricular function if the EF on echocardiogaphy is below 45%.

The sodium-channel blocker was changed during the study period: propafenone was replaced with flecainide, because the specific neonatal formula of propafenone was no longer available at our hospital.

With the antiarrhythmic treatment, we attempt either to suppress the arrhythmia or, if not possible, to achieve rate – control.

Blood pressure was monitored before and after the administration of propranolol. If blood presssure fell below age-related normal values, the dose was reduced. QRS complex duration was measured daily under the uptitration of sodium-channel blockers. Widening of the QRS complex by more than 20% of the baseline measurement resulted in dose reduction or cessation of the medication, if appropriate. Liver and thyroid function were checked at least monthly under amiodarone.

In general, the prophylactic antiarrhythmic treatment is maintained for one year and then discontinued. If recurrences occur, the medication is continued.

Primary end points of the study were freedom from arrhythmia, recurrence after one year of treatment, treatment-related complications, or death. We also analyzed the influence on outcome of fetal tachycardia, the type of tachycardia and the numbers of antiarrhythmic drugs.

## Statistics

Data are presented as frequencies, mean ± standard deviation (SD), or median and range as appropriate. Categorical data are given as frequencies. Continuous data were compared using the unpaired *t* test, and categorical data were compared using the Fisher’s exact test or the *χ*^2^ test with or without Yates correction as appropriate. A *p* value less than 0.05 defines statistical significance. The local and institutional ethics committee approved the study.

## Results

A total of 67 neonates and infants met the inclusion criteria. Patients’ characteristics at initial presentation and type of the SVT are summarized in Table 1. Four infants had a proven genetic abnormality (Rett syndrome, Braddock-Carey syndrome, Shwachman-Diamond syndrome, and cystic fibrosis in 1 patient each). A secundum-type atrial septal defect was present in 3, and a small muscular ventricular septal defect in 1 patient. 18% of the neonates were borne premature and none of them due to fetal tachycardia.

**Table 1:** Demographics and type of SVT at diagnosis

	Total	AVRT	FAT	AVNRT	P (AVRT vs FAT)
Number of patients (%)	67 (100)	48 (72)	18 (27)	1 (1)	
Male gender	34 (51)	23	11		0.5
Age (days)	14 (0–254)	11 (0–122)	22 (0–254)	184	0.22
Bodyweight (kg)	3.8 ± 1.4	3.7 ± 1.2	3.8 ± 1.6	7.5	0.84
Premature birth	12 (18%)	8	4		0.2
34–37 weeks	8	4	4		
30–34 weeks	3	3			
25–26 weeks	1	1			
Genetic abnormalities	4	1	3		0.06
Fetal SVT	18 (27%)	14	4		0.8
Fetal medical therapy	9	7	2		
Hospitals stay (days)	13 (0–166)	12 (3–166)	16 (0–51)	unknown	0.9

Genetic abnormalities: Rett, Braddock-Carey, and Shwachman-Diamond Syndrome and cystic fibrosis

*AVNRT* atrioventricular nodal re-entrant tachycardia, *AVRT* atrioventricular re-entrant tachycardia, *FAT* focal atrial tachycardia, *SVT* supraventricular tachycardia

*p* < 0.05 is considered statistically significant

Pre-excitation was detected on 12-lead ECGs in 6 patients during sinus rhythm. The ECG during tachycardia was indicative for AVRT in 48 (72%), including 1 with permanent junctional reciprocating tachycardia (PJRT), AVNRT in 1 (1%), and FAT in 18 (27%) patients, including 1 patient with multifocal atrial tachycardia.

A history of recurrent fetal tachycardia continuing postpartum was present in 18 (27%) neonates.

Intrauterine medical therapy was administered to 9 fetuses (4 received digoxin, 1 sotalol, 3 received a combination of digoxin and sotalol, and 1 a combination of digoxin and flecainide).

For the emergency treatment of the first episode of SVT, at least one acute maneuver to convert the tachycardia in sinus rhythm was performed in 47 (70%) patients; adenosine was administered intravenously to most of the patients (42/47). One patient with FAT needed ECMO support for 4 days due to cardiac and multiple organ failure caused by high-rate SVT despite medical therapy. Left ventricular (LV) function was classified as mildly impaired in 11 patients, moderately impaired in 5, and severely impaired in 1. After re-establishing sinus rhythm, LV function normalized in all patients.

Prophylactic medication was started in 65 (97%) patients and consisted of beta-receptor blocking agents in 42 (63%) (propranolol in 40 patients and atenolol and metoprolol in 1 patient each), propafenone in 20 (30%) patients, amiodarone in 20 (30%), and digoxin in 5 (8%). Two patients, 1 with AVRT and 1 with FAT, experienced only 1 episode of SVT and did not receive any treatment. First-hand single antiarrhythmic drug therapy was successful in controlling the tachycardia in 47 (70%) patients, 14 (21%) patients needed 2 antiarrhythmic drugs, and 4 (6%) patients needed 3 or more. Propafenone was discontinued in 7 patients due to widening of the QRS complex of more than 20% of the baseline measurement in 6 patients and a ventricular tachycardia in 1. Table 2 relates the medical therapy to the type of SVT. The effectiveness of beta-receptor blocking agent single therapy did not differ between AVRT and FAT (*p* = 0.09). In more than one-third of the patients with FAT (7/18), a single antiarrhythmic medication was insufficient to control the tachycardia.

**Table 2:** SVT mechanism and initially established medication

SVT mechanism	Prophylactic medication	*N* 67 (%)
AVRT		**48 (72)**
	Propranolol (metoprolol, atenolol in one patient each)	23
	Propafenone	8
	Amiodarone	5
	Propranolol + propafenone	4
	Propranolol + amiodarone	5
	Propranolol + amiodarone + digoxin	2
	No therapy	1
AVNRT		**1 (1)**
	Amiodarone	1
FAT		**18 (27)**
	Propranolol	4
	Propafenone	5
	Amiodarone	1
	Propranolol + propafenone	1
	Propranolol + amiodarone	1
	Propafenone + amiodarone	2
	Amiodarone + digoxin	1
	Amiodarone + digoxin + propranolol	2
	No therapy	1

*AVNRT* atrioventricular nodal re-entrant tachycardia, *AVRT* atrioventricular re-entrant tachycardia, *FAT* focal atrial tachycardia, *SVT* supraventricular tachycardia

Of the 18 neonates with a history of fetal tachycardia, 14 were diagnosed with AVRT; the remaining 4 patients had FAT. Prophylactic medication was started with the occurrence of the first postnatal tachycardia.

## Follow-up and Outcome

No SVT-related mortality occurred. Deaths due to an underlying disease occurred in 4 patients: 2 were affected by a syndrome and died of pulmonary infections and 2 due to respiratory failure: 1 due to chylothorax and 1 due to broncho-pulmonary dysplasia. Overall, 21 rehospitalizations occurred due to recurrences of tachycardia.

**Fig. 1:**
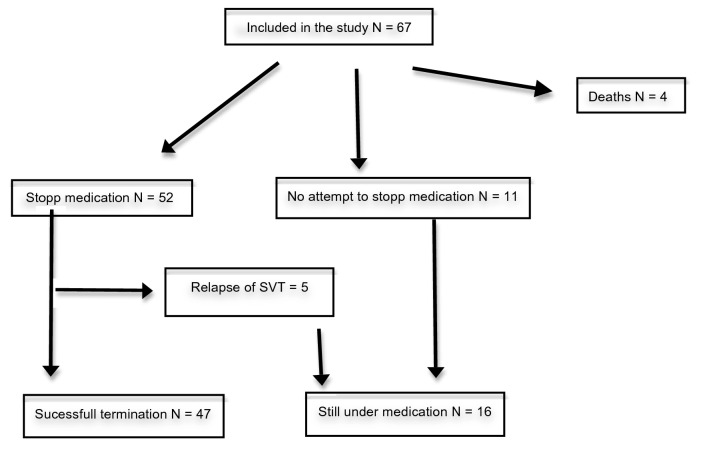
Flow chart of follow-up and outcome

The prophylactic medication was discontinued in 52 patients after a median time of 12 months (6–60), Fig. 1. Overall median follow-up thereafter was 23 months (5–114). In 5 patients, an SVT relapse occurred after a median time of 1 month (0–4), 2 out of these 5 had a history of fetal tachycardia. In 11 patients, no attempt was undertaken to withdraw the therapy, either because they were too young (attempted treatment period of 12 months not reached yet) or suffered too many tachycardia recurrences to allow such an attempt. In summary, successful termination of prophylactic treatment was possible in 47 (75%) of the surviving patients.

Table 3 shows the outcome after termination of the prophylactic medication. The prophylactic medication could be withdrawn in 33/41 (80%) of the infants receiving a single drug for prophylactic treatment. Double therapy could be stopped without relapse of the tachycardia in 10/16 (63%) and triple therapy in 2/4 (50%) patients.

**Table 3:** Final prophylactic antiarrhythmic medication

Number of drugs for prophylactic therapy	Medication	N	Still under prophylactic medication^a^ *N* (%)
Single-drug therapy		**41**	**8 (20)**
	Beta-receptor blocking agents (propranolol *N* = 23, metoprolol *N* = 1, atenolol *N* = 1)	25	2 (8)
	Propafenone	11	4 (36)
	Amiodarone	5	2 (40)
Dual-drug therapy		**16**	**6 (38)**
	Propafenone/flecainide + propranolol	8	2 (25)
	Amiodarone + propranolol	7	3 (43)
	Amiodarone + propafenone	3	1 (33)
Triple-drug therapy		**4**	**2 (50)**
	Amiodarone + digoxin + propranolol	3	2 (67)
	Amiodarone + propafenone + propranolol	1	0
No therapy		**2**	**0**

^a^No withdraw of medication possible or recurrence of SVT after cessation of medication

Most patients were free of arrhythmia after withdrawal of the prophylactic treatment: those with AVRT in 77% (36/47), those with FAT in 73% (11/15), Table 4

**Table 4:** Outcome in relation to type of tachycardia

SVT mechanism	N	Still under prophylactic medication^a^ *N* (%)
AVRT	47	11 (23)
AVNRT	1	1 (100)
FAT	15	4 (27)
Intrauterine SVT	17	10 (59)
No intrauterine SVT	46	6 (13)

*FAT* focal atrial tachycardia, *AVNRT* atrioventricular nodal re-entrant tachycardia, *AVRT* atrioventricular re-entrant tachycardia, *SVT* supraventricular tachycardia

^a^No withdraw of medication possible or recurrence of SVT after cessation of medication

Freedom from recurrence of arrhythmia after 1 year of treatment was not influenced by the number of drugs for prophylactic treatment (*p* = 0.16) or by the type of SVT (*p* = 1.0). In infants with episodes of fetal tachycardia withdrawal of prophylactic therapy was not possible in 10 patients (*p* = 0.007) due to persistent or recurrent tachycardias.

## Discussion

This study shows that prophylactic antiarrhythmic drug therapy for SVT in infancy used with our cohort is in the majority of cases safe and well tolerated. This is remarkable because complex pharmacokinetics and pharmacodynamics are anticipated in this age group, which are different from those in adults. Furthermore, milk and unsteady feeding schedules may interfere with drug absorption [15]. In many of the patients, antiarrhythmic treatment was initiated a few hours after birth in the delicate period of hemodynamic changes and organ adaptation to extrauterine life.

This study also confirms the therapeutic effect of the antiarrhythmic drugs used: Within the 10-year period analyzed, we were able to achieve either total suppression of the arrhythmia or a rate-controlled situation with only a few relapses in all patients in the study cohort. No tachycardia-related deaths were observed.

In our institution, the first-line medication in uncomplicated SVT is a beta-receptor blocking agent. In neonates and infants, we prefer propranolol because of wide experience with this medication [16], and the dosage can be adapted very well to the fast-changing weight in this age group. Propranolol was very well tolerated in our cohort. This finding is in line with the results of another large pediatric study in which propranolol was used to treat 287 neonates and infants with SVT: despite rather high doses of propranolol (median doses of 4 mg/kg/day) no serious side effects were seen. In particular, no hypotension or hypoglycemia occurred. The drug could be safely administered to all but 1 patient of the cohort, who experienced reversible bradycardia [17].

Sodium-channel blockers are highly efficient in treating SVTs in neonates and infants and have been used for decades [3, 15, 18]. They are known to influence the duration of the QRS complex, and proarrhythmic effects may occur. In our cohort, 7 of the initially treated 15 (47%) infants with sodium-channel blockers showed a significant (i.e. > 20% from baseline) prolongation of the QRS complex duration. One of these patients later experienced ventricular tachycardia.

A significant QRS widening (> 25% from baseline) in up to 50% of the treated patients, without leading to further proarrhythmic effects, was seen in 175 neonates and infants treated with flecainide. However, flecainide had to be discontinued in only 3% of the patients; this was due to cardiac dysfunction and proarrhythmia [18]. In contrast, no significant increases in the QRS complex occurred in 20 neonates treated with flecainide with a mean dose of 3.35 mg/kg/day [19]. This might be due to a flecainide dose at the lower limit.

Propafenone is frequently used in Europe, and it has proved to be an effective antiarrhythmic drug with low side effects [20–23]. Proarrhythmic events are observed in about 2% of patients treated [21, 22].

Since the dosage and product of sodium-channel blocker varies among studies, direct comparison is difficult; furthermore, the definition of adverse events is not consistent. In brief, we consider sodium-channel blockers effective for the treatment of SVTs in neonates and infants, but close monitoring of the QRS complex duration is mandatory as recommended [3] to prevent further proarrhythmic effects.

Digoxin is an old and effective antiarrhythmic drug. In some centers, it is the preferred drug for infants with SVT [6, 12, 16]. Its antiarrhythmic effects have been compared with propranolol in several studies: A multicenter, randomized controlled trial including 61 neonates and infants and a retrospective cohort study with 347 infants showed similar efficacy of propranolol and digoxin in the treatment of infants with SVTs [12, 16]. In contrast, another study of 484 hospitalized infants treated for SVT found that treatment failure and recurrence rate were more common on propranolol than digoxin [11]. However, hypotensive episodes, some even requiring inotropic support, occurred more often under digoxin [11]. Despite its efficacy, our institution only rarely uses digoxin today because of its narrow therapeutic window, which requires tight monitoring of serum levels, and potential harmful interactions.

The effectiveness of beta–receptor blocking agent therapy was not influenced by the electrophysiological properties of either FAT or AVRT tachycardia. Another study obtained corresponding results by treating all infant SVTs with high doses of propranolol; moreover, in this cohort, the type of arrhythmia was not associated with propranolol efficacy [17].

Prophylactic antiarrhythmic treatment could be successfully terminated in 75% of our patients after the planned treatment period of 12 months; thereafter the patients were free of arrhythmias. The proportion of successfully treated infants in our cohort is similar to other studies [5, 7, 9, 10].

In general, antiarrhythmic prophylaxis is recommended for a period of 12 months [4, 8, 12]. However, it might not always be necessary to maintain the antiarrhythmic therapy for 1 year, some infants might overgrow the tachycardia substrate faster and, therefore, be treated for a shorter time. In a cohort of infants with AVRT and AVNRT treated either with propranolol or digoxin, no first recurrence occurred after 4 months of treatment [12]. This implies that, in an uncomplicated SVT (i.e., no history of fetal tachycardia, prompt response to treatment, no recurrence of arrhythmia during medical therapy), termination of the treatment can be considered after a couple of months; however, this decision has to be made individually for each patient [4, 12].

In our cohort, no association was found between either the various medications or the SVT mechanisms and outcome. Furthermore, there was no difference in outcome between infants who had a single therapy or who needed more than one prophylactic medication to control the arrhythmia. This indicates that, even if initially the arrhythmia is difficult to control and the prophylactic treatment complex to adjust, this may not imply an unfavorable outcome. In contrast to our findings, a study conducted between 1971 and 1997 including 109 neonates with SVTs found that initial treatment difficulties were significant risk factors for prolonged arrhythmias [24]. However, these findings may not be directly comparable to our findings due to a cohort including more patients with pre-excitation (1/3 of the cohort) and different drug regimens (3/4 of the patients received digoxin).

In our cohort, infants with fetal tachycardias had a significantly higher risk of persistent tachycardias; 59% of the infants with a history of fetal tachycardia continued with antiarrhythmic treatment for more than one year. This result suggests that this group of patients may need longer prophylactic antiarrhythmic drug therapy. In a historic study with 17% fetal SVT in the cohort, fetal SVT was not identified as a risk factor for prolonged SVT problems [24]. Other authors also report favorable outcomes after fetal SVT, but always in small cohorts with 20–30 patients: 70–90% patients were asymptomatic without medication after the age of 1 year [25–27]. Some difference from our results might be explained by their inclusion of neonates with atrial flutter, who are known to have excellent prognosis. Furthermore, some groups give prophylactic antiarrhythmic treatment to all infants with a history of fetal tachycardia, whether or not the tachycardia persists postnatally.

## Limitations

This study has several limitations related to its observational and retrospective nature. The relatively small size of this sample minimizes the power of our statistical analysis. ICU admission and duration of hospital stay may not only be related to patients’ arrhythmia. There might be an information bias, as some infants were also treated in other hospitals according to their own treatment approach. Furthermore, SVTs, recurrence, and fetal occurrence may be unapparent, so the real number might be higher than observed in our study.

## Conclusion

Prophylactic antiarrhythmic medication for SVT in infancy is generally safe and well tolerated. Arrhythmia control is achieved with a single medication in the majority of patients. Initial administration of sodium-channel blockers such as propafenone or flecainide must occur under close monitoring of the QRS complex to prevent proarrhythmic effects. After cessation of the 1-year prophylactic antiarrhythmic treatment, most patients are free of recurrences. Infants with a history of fetal tachycardias are at risk for persistent tachycardias and recurrences. Our findings may contribute to generating specific guidelines for the treatment of SVT in neonates and infants. We speculate that in uncomplicated cases, the duration of treatment might be shortened, in contrast to infants with fetal onset of the tachycardia, in whom longer treatment might be beneficial. Further prospective controlled studies are required to evaluate the optimal duration of prophylactic treatment.

## Abbreviations

AVNRT: Atrioventricular nodal re-entrant tachycardia
AVRT: Atrioventricular re-entrant tachycardia
ECG: Electrocardiogram
EF: Ejection fraction
ECMO: Extracorporeal membrane oxygenation
FAT: Focal atrial tachycardia
LV: Left ventricle
PJRT: Permanent junctional reciprocating tachycardia
SVT: Supraventricular tachycardia
